# Unleashing the Potential of Nrf2: A Novel Therapeutic Target for Pulmonary Vascular Remodeling

**DOI:** 10.3390/antiox12111978

**Published:** 2023-11-07

**Authors:** Qin Fang, Yang Bai, Shuiqing Hu, Jie Ding, Lei Liu, Meiyan Dai, Jie Qiu, Lujin Wu, Xiaoquan Rao, Yan Wang

**Affiliations:** 1 Division of Cardiology, Department of Internal Medicine, Tongji Hospital, Tongji Medical College, Huazhong University of Science and Technology, Wuhan 430030, China; fangqin@tjh.tjmu.edu.cn (Q.F.); baiyang@tjh.tjmu.edu.cn (Y.B.); shuiqinghu@hust.edu.cn (S.H.); djdingjiewhu@163.com (J.D.); leiliu@tjh.tjmu.edu.cn (L.L.); daimylch@163.com (M.D.); tjqiujie@tjh.tjmu.edu.cn (J.Q.); lujinwu1990@tjh.tjmu.edu.cn (L.W.); 2 Hubei Key Laboratory of Genetics and Molecular Mechanisms of Cardiological Disorders, Huazhong University of Science and Technology, Wuhan 430030, China

**Keywords:** pulmonary vascular remodeling, Nrf2, oxidative stress, pulmonary hypertension

## Abstract

Pulmonary vascular remodeling, characterized by the thickening of all three layers of the blood vessel wall, plays a central role in the pathogenesis of pulmonary hypertension (PH). Despite the approval of several drugs for PH treatment, their long-term therapeutic effect remains unsatisfactory, as they mainly focus on vasodilation rather than addressing vascular remodeling. Therefore, there is an urgent need for novel therapeutic targets in the treatment of PH. Nuclear factor erythroid 2-related factor 2 (Nrf2) is a vital transcription factor that regulates endogenous antioxidant defense and emerges as a novel regulator of pulmonary vascular remodeling. Growing evidence has suggested an involvement of Nrf2 and its downstream transcriptional target in the process of pulmonary vascular remodeling. Pharmacologically targeting Nrf2 has demonstrated beneficial effects in various diseases, and several Nrf2 inducers are currently undergoing clinical trials. However, the exact potential and mechanism of Nrf2 as a therapeutic target in PH remain unknown. Thus, this review article aims to comprehensively explore the role and mechanism of Nrf2 in pulmonary vascular remodeling associated with PH. Additionally, we provide a summary of Nrf2 inducers that have shown therapeutic potential in addressing the underlying vascular remodeling processes in PH. Although Nrf2-related therapies hold great promise, further research is necessary before their clinical implementation can be fully realized.

## 1. Introduction

Pulmonary hypertension (PH) is a progressive and life-threatening disease, characterized by an excessive proliferation of pulmonary vascular cells, leading to pulmonary vascular resistance, oxidative stress, right ventricular hypertrophy (RVH), and eventually heart failure [1,2]. PH is defined by a pulmonary vascular resistance > 3 Wood units, pulmonary artery wedge pressure ≤ 15 mmHg, and an increase in mean pulmonary arterial pressure ≥ 20 mmHg at rest [3,4]. PH can be divided into five major categories: (1) pulmonary arterial hypertension (PAH), including heritable, idiopathic, and drug/toxin-induced PH; (2) PH due to interstitial lung diseases and/or hypoxia, such as chronic obstructive pulmonary disease and high-altitude; (3) PH induced by left heart disease; (4) chronic thromboembolic pulmonary hypertension; and (5) PH with unclear and/or multifactorial origin, including systemic and hematologic disorders [5,6]. PH most frequently occurs at a young age (30 to 60 years), with a morbidity of over 30–50 million individuals every year and a severely reduced life expectancy [7]. Disease progression is inevitable in most PH patients, and mortality remains unacceptably high despite appropriate treatment [8]. Upon diagnosis, the average life expectancy of PH patients is approximately 7–10 years [9].

Despite great progress having been made in the treatment of PH, no drugs have shown satisfactory efficacy so far [10]. Pharmacological agents currently authorized for the treatment of PH include soluble guanylate cyclase stimulators, endothelin receptor antagonists, phosphodiesterase inhibitors, and nitric oxide donors to prostacyclin analogs and prostacyclin receptor agonists. Even though these agents prompt pulmonary vasodilation and improve symptoms, they do not significantly improve long-term prognosis, primarily due to their inability to address the underlying pulmonary vascular remodeling (PVR) process [11,12]. As a result, novel therapies targeting the pathogenesis of PH are urgently warranted. Vascular remodeling, a central pathogenic process in all types of PH, is associated with the dysfunction of the endothelium, the proliferation and hypertrophy of vascular smooth muscle cells (VSMCs), and the accumulation of extracellular matrix [13,14,15]. These processes lead to neointima formation, medial hypertrophy, muscularization, and plexiform lesions’ development, contributing to the obliteration of precapillary pulmonary arteries and sustained elevation of pulmonary arterial pressure (PAH) [16,17]. Notably, the pathogenesis of PVR remains elusive. However, it is widely considered that oxidative stress is a crucial factor in PVR [18,19,20,21]. Oxidative stress induces several pathophysiological processes such as the dysfunction of pulmonary arterial endothelial cells (PAEC), excessive proliferation of pulmonary arterial smooth muscle cells (PASMC), extracellular matrix protein deposition, distal pulmonary arterioles muscularization, autoimmune processes, and inflammation [22,23], all of which are involved in the pathogenesis of PVR. Therefore, oxidative stress may be a potential target for the treatment of PH.

Nuclear factor erythroid 2-related factor 2 (Nrf2) is a key transcriptional factor involved in the amplification of the antioxidant pathways associated with cardiovascular diseases [24,25,26]. It protects the cells against oxidative stress from exogenous or endogenous factors via activating antioxidant genes [18,27,28]. Additionally, Nrf2-regulated pathways play various roles in the pathophysiology and physiology of endothelial cells (ECs), vascular smooth muscle cells, and extravascular cells, exerting antioxidative, anti-inflammatory, and cytoprotective effects [29,30,31]. Recently, several studies have shown that Nrf2 inducers have potent antivascular remodeling effects in animal models of PH. This review summarizes the mechanistic role of Nrf2 in PVR and discusses the preclinical evidence of the therapeutic potential of Nrf2 inducers in PH.

## 2. Molecular Basis of Nrf2

**Figure 1:**
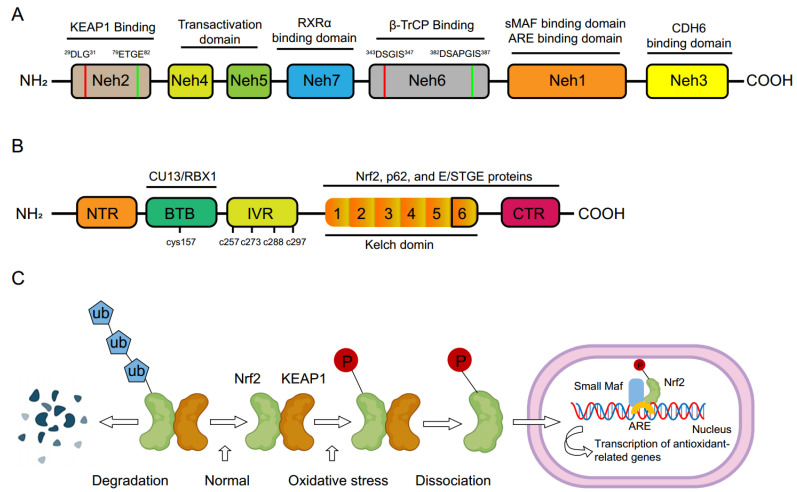
(**A**). Nrf2 protein comprises seven conserved area domains Neh1 to Neh7. Neh1 domain is a CNC-bZIP domain which allows Nrf2 to bind ARE through interaction with other factors like small musculoaponeurotic fibrosarcoma (sMAF). The Neh2 domain negatively controls the Nrf2 through its DLG and ETGE motifs. The Neh3 domain recruits chromo-ATPase/helicase DNA-binding protein family member CDH6. The Neh4 and Neh5 domains can interact with the CH3 domain of cyclic adenosine monophosphate (cAMP) response element binding protein (CREB)-binding protein. The Neh6 domain has two motifs, DSGIS and DSAPGS of β-transducin repeat-containing protein (β-TrCP). Neh7 domain interacts with retinoic X receptor alpha (RXR-α). (**B**). Keap1 protein comprises five domains such as N-terminal region (NTR), Bric-a-Brac domain (BTB), a cysteine-rich intervening region (IVR), Kelch domain, and carboxy-terminal region (CTR). Neh, Nrf2-ECH homology; CNC, cap ‘‘n’’ collar; bZIP, basic-region leucine zipper; Nrf2, nuclear factor E2-related factor 2; RXR-α, retinoid X receptor α; Keap1, Kelch-like ECH associated protein 1. (**C**). A scheme of the Nrf2 signaling pathway.

Nrf2 was first discovered in the homolog of the hematopoietic transcription factor p45 NF-E2 [32]. It is encoded by the gene Nfe2l2 and belongs to a member of the Cap‘n’Collar (CNC) family, which regulates over 250 genes that contain the antioxidant response element (ARE) sequence [33]. They constitute a defense response to oxidation factors, including genes that encode for HO-1, superoxide dismutase (SOD), glutamate-cysteine ligase (GCL), glutathione S-transferase (GST), thioredoxin reductase (TXNRD)1, thioredoxin (TXN)1, glutathione peroxidase (GPx), and lowered glutathione (GSH) [34,35,36]. Additionally, this family has six members, including Nf-e2, Nrf1, Nrf2, Nrf3, Bach1, and Bach2 [37]. Nrf2 has seven conserved areas named Nrf2-ECH homology domains, which consist of Neh1 to Neh7 (Figure 1A). Among them, the Neh1, Neh3 to 5 domains are related to the transcriptional activation of Nrf2 by binding its co-activators, while the Neh2, Neh6 to 7 domains control the stability of Nrf2 by responding as a negative regulatory domain [38]. Stabilized Nrf2 translocates to the nucleus and binds with ARE on DNA to activate genes’ transcription, thus reducing or eliminating the production of reactive oxygen species (ROS) [28,39,40].

## 3. Signaling Transduction of Nrf2

Nrf2 is regulated by two specific mechanisms: the Kelch-like ECH-associated protein 1 (Keap1) and the participation of β-TrCP with the glycogen synthase kinase-3β (GSK)-3β [41]. Keap1 is one of the main regulators of Nrf2 [42]. Keap1 consists of five functional domains, namely, the N-terminal (NTR) domain, Broad complex/Tramtrack, Bric-a-Brac domain (BTB), a cysteine-rich intervening region (IVR), Kelch domain, and carboxyterminal (CTR) domain (Figure 1B). These domains of Keap1 play important roles in mediating Nrf2 ubiquitination and repression. Keap1 regulates Nrf2 protein expression in a redox-dependent manner [43,44]. Nrf2 is ubiquitinated and degraded by the Keap1-Cul3-Rbx1 complex under normal redox conditions. Under stress conditions, highly reactive cysteine residues of Keap1 are oxidized, which disrupts the binding of Keap1 to Nrf2 and promotes the nuclear translocation of Nrf2 (Figure 1C). The formation of Nrf2-sMaf heterodimers via its Neh1 domain in the nucleus induces gene expression by binding to the ARE sequences in the promoter regions of Nrf2 target genes [45]. The Nrf2/ARE signaling pathway is the major pathway for intracellular redox balance. ARE-genes encode a broad network of enzymes involved in antioxidant mechanisms encompassing nicotinamide adenine dinucleotide phosphate (NADPH), phase I, II, and III biotransformation reactions, lipid and iron catabolism, glutathione and thioredoxin-mediated reactions, among others. These pleiotropic functions contribute to significant cardiovascular protection effects. In addition, glycogen synthase kinase-3 was shown to prompt Nrf2 degradation by phosphorylating Nrf2 at serine 335 and 338. The poly ubiquitination of Nrf2 and its recognition by β-TrCP increase Nrf2 degradation [40,46]. Nrf2 controls the basal and inducible expression of an array of antioxidant and detoxification enzymes including the proteasome (Table 1). The Nrf2 signaling system has emerged as perhaps the most vital cellular defense and survival pathway against oxidative stress and toxicants [47].

## 4. Role of Nrf2 in Pulmonary Vascular Remodeling

Pulmonary vascular remodeling (PVR) refers to the thickening of blood vessels in the lungs, which involves all three layers of the blood vessel wall including the intima, the media, and the adventitia. Various pathological conditions, including pulmonary hypertension and chronic lung diseases, may cause PVR, as manifested by the thickening, narrowing, and stiffening of pulmonary arteries, capillaries, and veins. This remodeling process involves endothelial dysfunction, smooth muscle cell phenotypic switching and proliferation, and alterations of the extracellular matrix. These structural changes lead to increased pulmonary vascular resistance, reduced blood flow, and impaired gas exchange, ultimately leading to symptoms such as shortness of breath and exercise intolerance. Emerging evidence indicates that Nrf2 and its downstream transcriptional targets are involved in multiple processes of PVR. Understanding the role of Nrf2 in PVR could provide important insights into potential therapeutic strategies for managing the condition of PH.

### 4.1. Nrf2 and Endothelial Dysfunction in the Intima Remodeling

Although the exact causes of abnormal vascular remodeling in PH are still under investigation, emerging evidence suggests that EC dysfunction is among the primary triggers that initiate this process. EC dysfunction causes the activation of a series of cellular signaling pathways, leading to the uncontrolled proliferation of PAECs, SMCs, and fibroblasts, and eventually gives rise to vascular remodeling and even the occlusion of the pulmonary vessels. When the ECs are damaged, the barrier function of the endothelium and the muscle–endothelium interface is destroyed, and the concomitant loss of ECs regulation of SMCs leads to increased SMC proliferation and pulmonary vascular reconstruction [48,49]. BMPR2 mutations, epithelial–mesenchymal transition (EMT), thrombus formation, apoptosis, and inflammation are other factors connected with ECs’ dysfunction in PAH [50,51,52]. BMPR2 is mainly present in ECs of the vascular lumen in the lung and its expression is decreased in ECs from PH patients. Hence, mutated BMPR2 is thought to play an important role in ECs’ dysfunction in PAH [53,54]. Recently, BMP9 is also reported to be involved in ECs’ dysfunction in PAH [55,56]. EMT is a phenomenon in which ECs lose endothelial markers, which is accompanied by the acquisition of a lot of mesenchymal-like phenotypes and an increase in mesenchymal markers. Moreover, ECs change their morphology by losing cell–cell contact and obtaining a highly invasive and migratory phenotype, therefore losing the features of a normal endothelium [57,58]. Endothelial dysfunctions are characterized by both an increased production of vasoconstrictors, such as thromboxane, serotonin, and endothelin-1(ET-1), and a reduced secretion of potent vasodilators, such as prostacyclin (PGI2) and nitric oxide (NO) [12,59]. Studies in animal experiments and patients have demonstrated that NO deficiency is involved in the pathogenesis of PH [60,61]. A decreased PGI2 level is detected in various patients with different forms of PH, partially explaining the increase in SMC proliferation and pulmonary vasoconstriction in these patients. In animal models with PH, overexpressing PGI2 synthase is protected from developing chronic hypoxia-induced PAH [62,63]. Endothelium secretion of ET-1 increases in PH. ET-1 is mainly synthesized in ECs and its highest level in the overall body is in the lungs. It exerts its effects by binding to the receptors of ETA and ETB, which activate PASMC regulating proliferation, vasoconstriction, and vasorelaxation [64,65,66]. Thromboxane A2, produced by platelets and ECs, is increased in PH [62,67]. It creates an imbalance that may lead to vascular remodeling and excessive platelet aggregation noticed in PH. ECs′ apoptosis can also play a part in PH development via vascular dropout and selection pressure, which gives rise to the apoptosis-resistant phenotype of ECs in vascular lesions [68].

**Figure 2:**
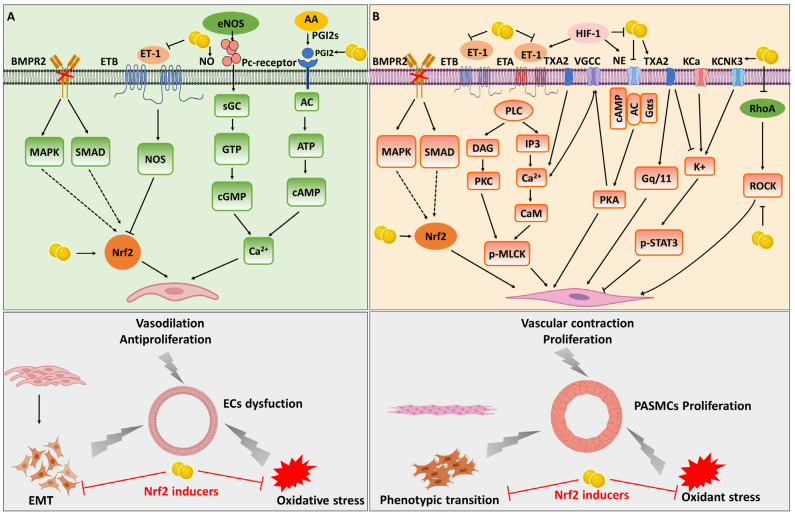
The mechanisms of activating of Nrf2 activity in PAEC dysfunction (**A**) and PASMC proliferation (**B**) in pulmonary hypertension. PAEC, pulmonary arterial endothelial cell; PASMC, pulmonary arterial smooth muscle cell.

ROS-induced oxidative damage aggravates ECs’ dysfunction. The oxidative stress inducers activate Nrf2 and then exhibit protective effects in ECs by inducing its downstream antioxidant genes (Figure 2A and Table 1). In addition, Nrf2 has angiogenic and anti-inflammatory functions in ECs. Animal studies have shown that increased oxidative stress due to Nrf2 knockout impairs endothelial function and reduces functional congestion [69]. Genetic Nrf2 knockout also exacerbates endothelial dysfunction in the aorta, brain, and skeletal muscle microcirculation induced by obesity [70,71]. Emerging evidence demonstrates that Nrf2 and its downstream targets are related to PH. Lin et al. showed that activating Nrf2 could suppress hypoxia-induced pro-inflammatory ET-1 and enhance the production of vasodilatation factor NO and PGI2 expression in ECs [72,73]. Ji et al. showed that Nrf2 could suppress the protein level of HIF-1α expression and was thought to be a critical transcription factor for controlling angiogenesis [74]. Nrf2 may also play a part in the regulation of BMPR2 in PH. Diebold et al. showed that decreasing BMPR2 in PAECs by siRNA during re-oxygenation decreased Nrf2 expression, mitochondrial membrane potential, and ATP while inducing mitochondrial DNA deletion and apoptosis [75]. Chen et al. showed that Nrf2 activation attenuated PVR by inhibiting EMT [76]. Recent studies showed that many agents such as Z-Ligustilide, Sulfasalazine and even long non-coding RNA (lncRNA), protected vascular ECs from oxidative stress in PAH by activating the Nrf2 signaling pathways [77,78,79]. HO-1, one of the primary targets of Nrf2 activation, is a critical endogenous antioxidant [80]. HO-1 can adjust EMT [76], which is shown to be involved in PH pathobiology [1,81]. Yet et al. demonstrated the important role of HO-1 in an animal model of PH [82]. They exposed HO-1^−/−^ mice to chronic hypoxia and verified that these mice developed right ventricular dilatation or failure and finally right ventricular infarcts. However, overexpressing HO-1 protected against hypoxia-induced lung inflammation and PAH [83]. A protective role of HO-1 was also documented in monocrotaline (MCT)-treated wild-type mice [84]. Furthermore, simvastatin could ameliorate PAH in MCT-treated rats via the induction of HO-1 expression [85]. Liang et al. also showed that bone-marrow-derived mesenchymal stem cells reduced chronic hypoxia-induced PAH via lung HO-1 expression [86]. In addition, HO-1 expression is substantial in lung tissue but decreased in the failing right ventricle in the Sugen 5416 and 10% chronic hypoxia (SuHx) model of PAH. Protandim, which can activate Nrf2 to upregulate the expression of genes encoding antioxidant enzymes, protects against the SuHx model of PAH in rats [87,88]. SOD is another downstream effector of Nrf2 activation. Hartney et al., who used pulmonary arteries acquired from calves with chronic hypoxic PAH, confirmed that the level of total SOD activity was reduced in the pulmonary arteries [89]. They showed that the overexpression of ECs SOD attenuated PH in mice. Van et al. also showed that overexpression of ECs SOD reversed bleomycin-induced lung fibrosis and nonangio-obliterative PH [90,91,92]. Additionally, SOD3, a subtype of SOD, is highly expressed in lung tissue [93]. Previous studies showed that the overexpression of SOD3 reduced MCT-induced PAH in rats and hypoxia-induced PAH in mice [94,95]. Xu et al. also showed that SOD3 defect in rats led to the development of PH, RVH, and vascular remodeling in response to hypoxia or MCT [96]. However, further experiments using endothelial or smooth-muscle-cell-specific knockout of SOD3 may be needed to investigate the cell-specific role of SOD3 in PAH. These results demonstrate that HO-1 and SOD, as vital downstream effectors of Nrf2, play crucial roles in the development of PH.

### 4.2. Nrf2 and Smooth Muscle Cell Phenotypic Switching in Media Remodeling

Rabinovitch et al. showed that BMPR2 mutations lower BMP signaling, contributing to the loss of the antiproliferative effects of BMP2 in PASMCs from PH patients [97]. In addition, ET-1 binds to the receptors of ETA and ETB, leading to complicated signaling pathways [98], involving the lowering of Potassium (K^+^) channel expression and activity, activation of Rho kinase (ROCK), and up-regulation of the [Ca^2+^] channel and Na^+^/H^+^ exchanger (NHE) [99,100]. Previous research showed that ET receptor inhibitors restrained or reversed vascular remodeling and PH in some animal models [101]. Thromboxane A2(TXA2) leads PH by inhibiting potassium channels, binding specific Gq/11 protein, and increasing intracellular calcium concentration ([Ca^2+^]i) [102,103,104]. It is revealed that PASMCs from PH patients were depolarized [105]. Reduced K^+^ channel activity and expression were subsequently confirmed as contributing factors to depolarizing PASMCs in hypoxic PH [106,107,108]. PASMCs express several K^+^ channel families, such as calcium-sensitive K(Ca), inward rectifier (Kir), voltage-gated (K(v)), ATP-sensitive K^+^ channel (KATP), and two-pore channels (K_2P_). Increasing evidence demonstrated that augmented K^+^ channel activity or expression lowered hypoxia-induced remodeling in animals and increased apoptosis in PASMCs from PH patients [109,110]. K(Ca) channels in VSMCs are targets for various physiological factors released from the endothelium, including NO and endothelium-derived hyperpolarizing factor (EDHF) [111,112]. The activation of voltage-gated calcium channels (VGCC) by agonists leads to PASMC proliferation [113,114]. Kir channels were first described in the SMCs of the coronary artery and were thought to express preferentially in small rather than large arteries [115]. KATP channels have numerous roles in PASMCs. They play an important role in maintaining membrane potential. Furthermore, the closure of KATP channels up-regulates the Ca^2+^ concentration in the cytoplasm, thus promoting the proliferation and contraction of PASMCs [116]. On the contrary, the opening of KATP channels results in an increase in membrane hyperpolarization, K^+^ efflux, inhibition of Ca^2+^ influx, and relaxation of the PASMC. Additionally, the EDHF and the NO can also activate KATP channels [117]. The K_2P_, also called potassium channel subfamily K member (KCNK), is a K^+^ channel in the subfamily K. It gathers different sub-families which are composed of several members [118]. KCNK3 is expressed in PASMC in humans. Furthermore, the knockdown of KCNK3 induced an obvious depolarization of resting membrane potential, demonstrating the significance of the KCNK3 channel in human PASMC resting membrane potential [119]. Increased [Ca^2+^], which has been verified in monocrotaline (MCT) and hypoxic animals treated with PH, is necessary for PASMC migration and growth. Additionally, the NHE is the primary contributor to the maintenance of PASMC pH homeostasis [120]. NHE activity is related to growth factor-induced proliferation. Lowering NHE activity via genetic deletion or pharmacological inhibition reduced hypoxia-induced vascular remodeling and PASMC migration and proliferation [121,122,123]. In addition, animal studies uncovered the role of HIF-1 in the development of hypoxia-induced PH [124,125,126]. Vascular remodeling and PH were improved in chronic hypoxia-stimulated Hif1a^+/−^ mice, while hypoxia-induced proliferation in PASMCs extracted from Hif1a^+/−^ mice was alleviated [124].

Pulmonary artery remodeling and vasoconstriction are key factors responsible for the vascular resistance observed in PH patients [127]. VSMCs’ excessive proliferation, hypertrophy, and apoptosis lead to the formation of the characteristic angio-proliferative lesions found in PH [128]. Oxidative stress plays a vital role in VSMC’s structure and function, and Nrf2 is one of the main antioxidant systems [129]. The expression of Nrf2 in rodent models of primary or secondary PH is decreased [130]. The selective delivery of Nrf2 activators to the injured vasculature has the potential to attenuate oxidative stress and decrease VSMC hyper-proliferation and migration towards the inner vessel wall [131]. Nrf2 gene transfer or Nrf2-inducing drugs such as sulforaphane, epigallocatechin gallate, cinnamic aldehyde, and exendin-4 have shown therapeutic applications in vascular diseases [132,133,134,135,136]. Previous research showed that the Nrf2/HO-1 pathway protected VSMCs from oxidative stress damage [137]. Another study showed that the activation of Nrf2 signaling alleviated VSMC phenotypic switching and vascular remodeling [138]. Recently, He et al. described a novel mechanism in which Nrf2 is a key regulator of VSMCs’ phenotypic switching and demonstrated a direct role for Nrf2 in the phenotypic switching of VSMCs during vascular remodeling [138]. Nrf2 exerts protective effects against PH development by mediating signaling pathways involved in their proliferation, migration, apoptosis, and phenotypic transition (Figure 2B). Of note, a previous study also showed that the antioxidant effects, which were mediated via the activation of the NO-cGMP-PKG-KATP channel signaling, relied on the Nrf2/HO-1 pathway [139]. Ko et al. showed that Nrf2 could suppress cell motility through RhoA-ROCK1 signaling [140]. Importantly, fasudil, a Rho-kinase inhibitor, mitigates pressure-overload-induced heart failure by activating Nrf2-mediated antioxidant responses [141]. This evidence demonstrates that Nrf2 is involved in the regulation of multiple signaling pathways of the occurrence and development of PH.

Pulmonary artery tension is mainly regulated by PASMC resting membrane potential and pulmonary artery endothelial function [142]. K^+^ channels are key regulators of vascular tone, cell proliferation, and apoptosis rates in PAH. PASMCs express several K^+^ channel families, including calcium-sensitive K(Ca), voltage-gated (K(v)), inward rectifier (Kir), ATP-sensitive K^+^channel (KATP), and K_2P_. The modulation of K^+^ channel activities by cellular oxidative stress has emerged as a significant determinant of vasomotor function in multiple disease states. Oxidative stress impairs K(v) channel function in persistent PH. Superoxide scavengers may improve pulmonary vasodilation in persistent PH of newborns in part by restoring K(v) channel function [143]. K(v) channel expression declined in PAH, resulting in elevations in cytosolic K^+^ and Ca^2+^ that decrease apoptosis and increase proliferation [144]. Pozeg et al. showed that K(v) in PASMCs is one of the reasons that led to pulmonary vasoconstriction even in PH [110]. K(v) channel activation significantly weakened the development of chronic hypoxia-induced PAH in mice and reversed spontaneous PAH [145]. In addition, during the formation process of left-to-right shunt-induced PAH, the function of the K(v) channel was inhibited, suggesting that K(v) channel may be the mechanism of PAH induced by left-to-right shunting [146]. One of the notable physiological functions of sequestosome1/p62 (SQSTM1) is the regulation of redox-sensitive voltage-gated potassium K(v) channels. Previous research confirmed that SQSTM1 enhanced the phosphorylation of K(v), which induced the suppression of pulmonary arterial Kv1.5 channels under acute hypoxia [147]. However, SQSTM1, regulated by the redox-sensitive transcription factor Nrf2, is an oxidative-stress-inducible protein. Therefore, Nrf2 may alleviate PAH by regulating K(v) channels. Cornfield et al. confirmed that reduced K(Ca) channel gene expression might lead to abnormal pulmonary vascular reactivity related to the persistent PH of the newborn [148]. Nrf2 is a vital determinant of the K(Ca) channels’ function and expression in VSMCs. Nrf2 promotes the expression of K(Ca) channels via a direct increase in gene transcriptions or other mechanisms [149]. Nrf2 is reported to be involved in KCNK3-regulated PAH vascular remodeling. KCNK3 expression and activity are strongly reduced in PASMCs in the PAH animal model. KCNK3 inhibition increased vasoconstriction, PASMC proliferation, and inflammation. Pharmacological activation of KCNK3 mitigated MCT-induced PAH in vivo [150]. Of note, Antigny et al. showed that the loss of function mutation of KCNK3 was responsible for the first channelopathy identified in PAH. They showed that the loss of KCNK3 expression resulted in the activation of the Nrf2/HO-1-mediated antioxidative stress response [151]. These results demonstrated that the loss of KCNK3 impaired PASMC function and a compensatory increase in Nrf2 could protect against it. Iptakalim (Ipt) is a new selective K(ATP) channel opener via electrophysiological, pharmacological, receptor binding tests and biochemical studies. In hypoxia-induced animal models, Ipt reduces the elevated mean pressure in pulmonary arteries and alleviates remodeling in the airways, right ventricle, and pulmonary arteries. Jin et al. showed that Ipt suppressed the effects of ET-1, decreased the intracellular calcium concentration, and restrained the proliferation of PASMCs [152]. Moreover, both the efficacy and safety of Ipt have been demonstrated in experimental animal models as well as in phase I clinical trials. Therefore, Ipt could be a potential candidate for hypoxic PH in the future [152]. Of note, Ipt protects against stress-induced oxidative stress through Nrf2-related pathways [153].

## 5. The Therapeutic Potential of Nrf2 Inducers in PH

In this section, we summarize the current potential therapeutic inducers targeting Nrf2 (Table 2) and discuss their potential applications for PH in preclinical studies.

### 5.1. SFN

SFN is the most widely used Nrf2 inducer in preclinical studies [190]. In addition to promoting the expression of endogenous antioxidants to regulate oxidative stress [191], it has significant anti-inflammatory effects and promotes cardiac protection in preclinical diabetic models. It has been reported that SFN protects against lung injury by activating the Nrf2 signaling pathway. Moreover, hypoxia-induced oxidation stress and pulmonary injury were significantly reduced by SFN in Nrf2^+/+^ mice but not in Nrf2^−/−^ mice [192]. Zhang et al. showed that SFN prevented pulmonary damage by activating the Nrf2-defense response and subsequently ameliorating inflammatory responses by modulating cytokine production [193]. Keller et al. showed that SFN inhibited right ventricular injury and decreased PVR in PAH [154]. Male mice induced with SuHx for the PAH model were randomized to SFN treatment at a daily dose of 0.5 mg/kg, 5 days per week, for 4 weeks. The data showed that SFN reduced SuHx-induced PVR, fibrosis, and inflammation by upregulating Nrf2 expression. It also prevented SuHx-induced right ventricular dysfunction and remodeling. Another study demonstrated that the effect of SFN on vascular remodeling depends on Nrf2 [155]. Wild-type (WT) and Nrf2 knockout mice were induced by SuHx for PAH, followed by treatment with or without SFN for 4 weeks. The results showed that SFN partially or completely reversed SuHx-induced RV systolic/diastolic dysfunction in the WT mice, but not in the Nrf2 knockout mice. Although the role of SFN in PH has not been verified in clinical studies, these data suggest that SFN may be a candidate to prevent PAH. Very importantly, SFN has shown good tolerability, safety, and efficacy in clinical applications such as autism spectrum disorder [194].

### 5.2. Oltipraz

Oltipraz, an agonist of Nrf2, exerts a strong effect against oxidative stress in animal models or clinical patients with certain diseases [195]. Oltipraz can increase the binding activity of Nrf2 to the antioxidant response element, thereby increasing the production of phase II enzyme genes [196,197]. Induction of the expression of SOD and HO-1, downstream effectors of Nrf2, via Oltipraz administration in an animal model of chronic hypoxia suggests that Oltipraz may exert its beneficial effect via Nrf2 activation. Eba et al. showed that Oltipraz significantly reduced PVR and RVH associated with chronic hypoxia-induced PH [156]. They exposed Nrf2-deficient mice and Keap1 knockdown mice to hypoxia, and then treated the mice with different doses of Oltipraz (5, 50, or 500 mg/kg). They found that Nrf2-deficient mice exposed to hypoxia developed more pronounced RVH than WT mice, while Keap1-knockdown mice exposed to hypoxia displayed less PVR and RVH than WT mice, emphasizing the beneficial potency of Nrf2 activity against PH. They also showed a decreased expression of Nrf2-regulated antioxidant enzymes in chronic obstructive pulmonary disease-related PH patients. Moreover, pharmacologically inducing Nrf2 activity with Oltipraz significantly reduced PVR and RVH in the hypoxia-induced PH model. These data showed that Nrf2 activation exerted therapeutic efficacy against hypoxia-induced PH. Of note, the efficacy of Oltipraz highlights the therapeutic potential of Nrf2 activators for PH prevention in patients with hypoxic lung disease. However, further clinical studies are still needed to demonstrate the therapeutic potential of Oltipraz in PH.

### 5.3. Resveratrol

Resveratrol is a natural polyphenol found in red wine and grape skins. It has diverse biochemical and physiological actions including antiproliferative properties. It has been reported that treatment with resveratrol could restore the natural compound activator of Nrf2 and thus decrease oxidative stress [198]. Several studies have validated the protective role of resveratrol in animal models of PAH [157,158,159,160]. The role of Nrf2 induced by resveratrol in PAH has been extensively reviewed elsewhere [199].

### 5.4. Rosiglitazone

Rosiglitazone is an antidiabetic agent that belongs to the thiazolidinediones and is a nuclear hormone receptor peroxisome proliferator gamma (PPARγ) agonist [200,201]. Many studies showed that rosiglitazone alleviated hypoxia-induced pulmonary arterial remodeling [166,202], and an inhaled combination of sildenafil and rosiglitazone improved cardiac function, pulmonary hemodynamics, and arterial remodeling [203]. Rosiglitazone alleviated ET-1-induced vasoconstriction of pulmonary arteries in the rat model of PAH via the differential regulation of ET-1 receptors [167]. Previous studies showed that the expression of PPARγ was decreased in the lungs of PH patients, and PPARγ ligands were related to the release of vasoactive substances from vascular ECs and prevention of vascular remodeling. In a rat model of hypoxia-induced PH, oral administration of rosiglitazone (8 mg/kg, 5 days/week) for 4 weeks significantly reduced PVR and the development of PH. Rosiglitazone treatment restrained the hypoxia-induced reduction in PPARγ expression and restored VEGF and ET-1 expression almost to the levels of the normoxia group [168]. At the same time, another study also showed that rosiglitazone (10 mg/kg/d) prevented the development of PH at 3 weeks, and reversed established PH at 5 weeks [169]. Moreover, Wang et al. showed that the protective effects of rosiglitazone against MCT-induced PH were associated with drug dose and might be due to the inhibition of inflammation. In a rat model of MCT-induced PH, the administration of rosiglitazone (2.5 and 5 mg/kg) significantly attenuated the perivascular inflammation in the PH group. Compared with the low-dose rosiglitazone intervention group, all the above indices of the high-dose rosiglitazone intervention group appeared much lower [170]. Importantly, many studies showed that rosiglitazone was an activator of Nrf2 [204,205,206]. However, it is not clear whether the protecting effect of rosiglitazone against PH relies on Nrf2. Further studies are warranted to validate the role of Nrf2 in the therapeutic potential of rosiglitazone in PH. Considering that rosiglitazone is an FDA-approved treatment for diabetes, the preclinical studies of PH are expected to translate quickly into clinical trials.

### 5.5. Dimethyl Fumarate

Dimethyl Fumarate (DMF) is an FDA-approved antioxidative agent with good security. More and more data have confirmed that DMF is a powerful activator of Nrf2 [207,208]. Grzegorzewska et al. showed that DMF therapy is effective in reversing hemodynamic changes and reducing oxidative damage and lung fibrosis in the experimental models of PAH. They showed that DMF had pleiotropic modes of action that might improve PAH. DMF not only prevented the development of increased right ventricular systolic pressure (RVSP) and RVH in SuHx- and hypoxia-induced mice but also reversed pre-existing PH in a chronic hypoxia animal model [172]. Muralidharan et al. provided a rational design of advanced inhalable therapeutic dry powders containing DMF to treat pulmonary inflammation [209]. These data substantiate the beneficial effects of DMF on important molecular pathways leading to PAHs and support the treatment of patients with PAH. They also demonstrated a novel form of Nrf2 agonist for PH treatment, which could significantly reduce systemic side effects.

### 5.6. Rutin

Rutin is a bioflavonoid with a wide range of biological activities, including antioxidant and anti-inflammatory effects. Rutin reduced oxidative stress, vascular fibrosis, and cardiac hypertrophy by upregulating Nrf2 transcriptional activity and the expression levels of its downstream target genes [210]. Previous studies showed that rutin protected against lung inflammation and lung injury [211,212,213]. Bai et al. demonstrated that rutin could protect against bleomycin-induced lung fibrosis [212]. Li et al. showed that rutin also increased NO production in bovine PAECs, and exerts a vasodilator influence on the pulmonary artery [214]. In addition, rutin attenuated hypoxia-induced PASMC proliferation [215]. However, Shellenberger et al. showed that although rutin reduced the severity of hypoxia-induced PH, histological changes consistent with pulmonary arteriole remodeling were observed in some calves fed rutin. They used calves fed rutin under hypoxic conditions, which had lower mean PAH. Paradoxically, a larger proportion of calves fed rutin had more histological evidence of pulmonary arteriolar adventitial hyperplasia and medial hypertrophy than the controls did [173]. The controversial data may be due to the use of different experimental methods. These also suggest that rutin may play different roles in different cells or tissues, which requires further research.

### 5.7. Curcumin

Curcumin, a lipophilic polyphenol, has anticancer, antibiotic, anti-inflammatory, and antioxidant effects, as suggested by several experimental studies and clinical trials. Several studies have shown that curcumin may protect lungs from oxidative damage by activating the Nrf2 signaling pathway [216,217]. These have shown that curcumin could improve PAH, ameliorate pulmonary vessel remodeling, and reduce the deposition of collagen I in pulmonary arterioles [175]. Devadasu et al. attempted to exert the therapeutic benefits of curcumin in PH by encapsulating it in biodegradable polyacid nanoparticles. However, orally administered nanoparticulate curcumin failed to provide any protection against hypoxia-induced PH, and changes in RVH and vascular remodeling were similar to those in the untreated group. They found an obvious difference in the target tissue levels between normoxic and hypoxic rats. The target tissue levels of curcumin under hypoxia are much lower than those in normoxic rats. This might be due to the difference in particle dynamics, leading to the failure of treatment [218]. Kruangtip et al. showed that curcumin analogues were potential targets for developing efficacious and selective PDE5 inhibitors and treatment of PH [219]. Chen et al. suggested that curcumin could promote PASMC apoptosis, protect mitochondrial function, and reverse MCT-mediated PVR. It could suppress the PI3K/AKT pathway in PASMCs and regulate the expression of antiproliferative genes [174]. However, they also reported the side effects of curcumin in MCT-induced rats, which reminds us that the dosage should be used with caution and its toxicological mechanism should be further evaluated and studied.

### 5.8. Natural Products

Puerarin is primarily derived from Gegen (Pueraria lobata, Radix puerariae) and exhibits a variety of pharmacological activities by activating the Nrf2 signaling pathway [220,221]. Previous studies have demonstrated that puerarin prevents hypoxia-induced PVR and PH [177,178]. Li et al. showed that puerarin (20 mg/kg/d, 3 weeks) could improve PVR in rats with PH by inhibiting the deposition of collagen. They showed that puerarin increased NO and SOD, and reduced ET-1 and collagen I levels [176]. Tannins present in Terminalia bellirica have pharmacological activities such as oxidative stress resistance and anti-inflammatory effects [222,223]. Recent research has shown that it might reduce high-altitude PH by activating the Nrf2/HO-1 signaling pathway in rats [222]. Naringenin, a flavanone extracted from various plants, has potent vaso-protective effects likely related to the induction of Nrf2 and HO-1 [224]. Ahmed et al. showed that naringenin significantly added to the protective effect of L-arginine in PH induced by MCT in rats. The mechanism might be to increase the expression of eNOS and the expression of NO and to reduce the expression of iNOS [180]. Berberine is an isoquinone alkaloid which is derived from berberis aristata, berberis aquifolium and berberis vulgaris. It has all kinds of pharmacological effects, such as antioxidant, antitumor, antidiabetic, anti-inflammatory, and antimicrobial. Several studies have shown that the biological and therapeutic activities of berberine depend on Nrf2 activation [225,226]. Yu et al. also showed that it alleviated PH through the β-catenin and Trx1 signaling pathways in PASMCs [181]. Tanshinone IIA is the major antioxidant component in salvia miltiorrhiza. Nrf2 activation is the major regulatory pathway in tanshinone IIA-induced cytoprotective gene expression against oxidative stress and inhibition of SMCs proliferation [227]. Previous studies showed that tanshinone IIA ameliorated hypoxic PH by modulating K(v) currents and inhibited Ca^2+^ influx [189,228]. Another study suggested that tanshinone IIA markedly decreased right ventricular systolic pressure, mean RVSP, RVH index, and PVR in MCT-induced PH rats [184]. However, whether these natural products can inhibit the development of PH by directly acting on Nrf2 remains to be verified.

## 6. Concluding Remarks and Future Perspective

We discussed and summarized the therapeutic potential of Nrf2 inducers for PAH in preclinical studies in this article (Table 2). It is demonstrated that the activation of Nrf2 inhibits vascular remodeling and PH mainly by improving ROS-related pathological processes. Moreover, we found some knowledge gaps. Firstly, we do not know whether Nrf2 inducers have the same effects in other vascular remodeling-related diseases, such as aortic dissection and chronic obstructive pulmonary disease. Secondly, although some Nrf2 inducers have been reported to improve PVR and PH, it is not clear whether they act directly through Nrf2. Therefore, further studies are needed to validate their effect by using genetically Nrf2-deficient mice. Thirdly, the Nrf2 inducers used in these studies are not specific to Nrf2. Thus, transgenic mouse models, specific Nrf2 inducers, and cell-specific overexpression or knockout of Nrf2 should be used to dissect the role of Nrf2 in PH. Finally, although many preclinical studies have confirmed the treatment effect of Nrf2 inducer in PH, it has not been verified via clinical trials. Considering that some of the Nrf2 inducers such as rosiglitazone and DMF have already been approved by the FDA for clinical use in other diseases, they may be quickly translated into the clinic to explore the effect on PH. It is encouraging to note that these Nrf2 inducers have shown good efficacy, tolerability, and safety in other diseases such as neurodegenerative disease [229]. Taken together, Nrf2-based PH therapy shows great promise to address the underlying vascular remodeling processes in PH, but the real treatment effect by targeting Nrf2 specifically and the exact molecular mechanisms need to be further studied. Further research in this area may pave the way for the development of innovative treatment options for PH patients.

## Figures and Tables

**Table 1:** A list of Nrf2 target genes about antioxidants.

Function	Gene	Extended Name
	*CAT*	catalase
	*GCLC*	glutamate-cysteine ligase catalytic subunit
	*GCLM*	glutamate-cysteine ligase modifier subunit
	*GGT1*	gamma-glutamyltransferase 1
	*GPX1*	glutathione peroxidase 1
	*GPX2*	glutathione peroxidase 2
	*GPX4*	glutathione peroxidase 4
	*GSR1*	glutathione reductase
Antioxidants	*HO-1*	heme oxygenase 1
	*NQO1*	NAD(P)H quinone dehydrogenase 1
	*PRDX1*	peroxiredoxin 1
	*PRDX6*	peroxiredoxin 6
	*SLC7A11*	solute carrier family 7 member 11
	*SOD*	superoxide dismutase
	*SRXN1*	sulfiredoxin 1
	*TXN1*	thioredoxin 1
	*TXNRD1*	thioredoxin reductase 1

**Table 2:** Therapeutic potential of Nrf2 inducers in pulmonary vascular remodeling in pulmonary hypertension.

Compound	Chemical Structure	Model	Treatment Strategy	Effects	References
SFN	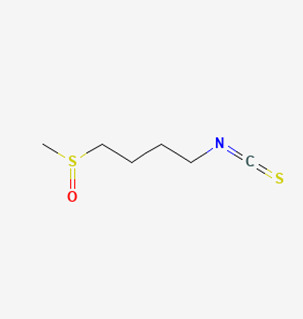	SuHx in mice	SFN (0.5 mg/kg 5 days per week) for 28 days	Prevented SuHx-induced RV dysfunction and remodeling, reduced RV inflammation and fibrosis, reduced SuHx-induced pulmonary vascular remodeling, inflammation, and fibrosis.	[154]
		global Nrf2-knockout mice, SuHx in mice	SFN (0.5 mg/kg 5 days per week) for 28 days	Partially or completely reversed SuHx-induced RV diastolic/systolic dysfunction and increased RV systolic pressure.	[155]
Oltipraz	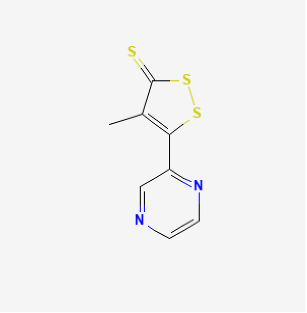	Hypoxia mice	Oltipraz (5, 50, or 500 mg/kg/day) for 3 days	Decreased RVH and pulmonary vascular remodeling.	[156]
Resveratrol	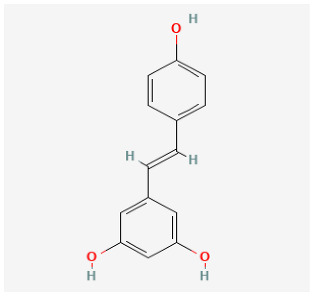	MCT rat	resveratrol (3 mg/kg/day) for 14 days	Attenuated established MCT-induced PH indices, including right ventricular systolic pressure, right ventricular hypertrophy, and medial thickening of intrapulmonary.	[157]
		MCT rat	resveratrol (10 and 30 mg/kg) twice daily for 21 days.	Attenuated RV hypertrophy, swollen mitochrondria and cardiomyocyte apoptosis.	[158]
		MCT rat	resveratrol (25 mg/kg/day) for 21 days.	Reduced the thickness of the pulmonary trunk tunica media.	[159]
		MCT rat	resveratrol (25 mg/kg/day) for 21 days	Exerted anti-inflammatory, antioxidant, and antiproliferative effects.	[160]
		Hypoxia rat	resveratrol (40 mg/kg/day) for 28 days	Prevented pulmonary hypertension through its antiproliferation, anti-inflammation and antioxidant effects.	[161]
		Hypoxia rat	resveratrol (40 mg/kg/day) for 21 days	Prevented pulmonary hypertension and RVH.	[162]
		Hypoxia rat	resveratrol (100 mg/kg/day) for 14 days	Prevented proliferation of human pulmonary artery smooth muscle cells and RVH.	[163]
		MCT rat	resveratrol (25 mg/kg/day) for 28 days	Prevented pulmonary vascular remodeling.	[164]
		MCT rat	resveratrol (25 mg/kg/day) for 28 days	Inhibited pulmonary arterial remodeling.	[165]
Rosiglitazone	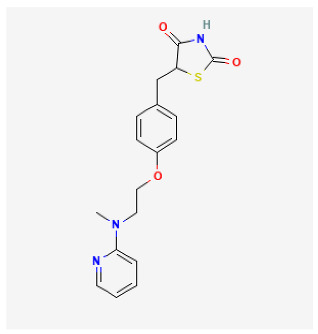	Pregnant rats were treated with nitrofen	Rosiglitazone (3 mg/kg/day) for 3 days	Reduced pulmonary vascular remodeling.	[166]
		Hypoxia rat	rosiglitazone (20 mg/kg per day) with oral gavage for 3 days	Inhibited pulmonary artery vasoconstrictive.	[167]
		Hypoxia rat	rosiglitazone (8 mg/kg orally, 5 days/week) for 28 days	Reduced chronic hypoxic pulmonary hypertension.	[168]
		Hypoxia mice	rosiglitazone (10 mg/kg/d, gavage) for 35 days	Reduced pulmonary vascular remodeling and hypertension.	[169]
		MCT rat	rosiglitazone (5, 2.5 mg/kg/day) for 21 days	Reduced perivascular inflammation.	[170]
		Hypoxia rat	rosiglitazone (5 mg/kg/day) for 21 days	Attenuated hypoxia-induced pulmonary arterial remodeling.	[171]
DMF	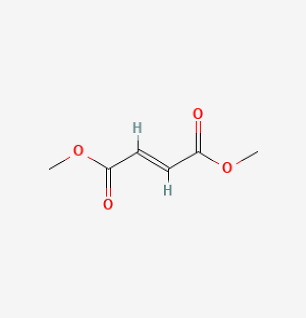	Hypoxia mice	DMF (90 mg/kg/day) for 21 days	Reversed hemodynamic changes, reducing inflammation, oxidative damage, and fibrosis.	[172]
Rutin	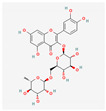	Hypoxia calves	glucorhamnoside rutin orally administered for 14 days	Led to pulmonary arteriolar medial hypertrophy and adventitial hyperplasia.	[173]
Curcumin	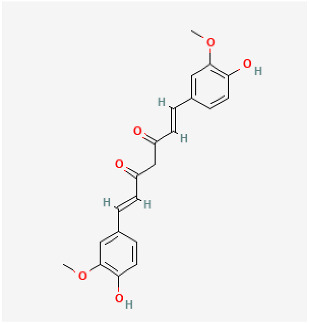	MCT rat	curcumin (30 mg/kg/day) for 18 days	Improved pulmonary vascular remodeling, promote PASMC apoptosis, and protect mitochondrial function.	[174]
		Hypoxia rat	curcumin (50 mg/kg/day) administrated for 28 days	Decreased pulmonary arterial pressure, improve pulmonary vessel remodeling and inhibit the deposition of collagen I in pulmonary arterioles.	[175]
Puerarin	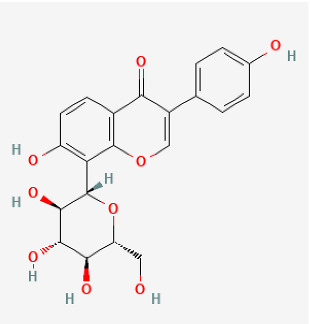	Hypoxia rat	puerarin (20 mg/kg/day) for 21 days	Improved pulmonary vascular remodeling.	[176]
		Hypoxia rat	puerarin intraperitoneal injection, 20 mg/kg/d for 21 days	Inhibition of vascular wall thickening pulmonary fibrosis.	[177]
		Hypoxia rat	puerarin (80 mg/kg/day, orally) for 21 days	Reduced autophagy and suppressing cell proliferation.	[178]
		MCT rat or Hypoxia mice	puerarin (10, 30, 100 mg/kg/d, i.g.) for 28 days or puerarin (60 mg/kg/d, i.g.) for 7 days	Reduced RVSP and lung injury, improved pulmonary artery blood flow, inhibit inflammatory responses, improved resistance to apoptosis and abnormal proliferation, attenuate right ventricular injury and remodeling, and maintained normal function of the right ventricle.	[179]
Naringenin	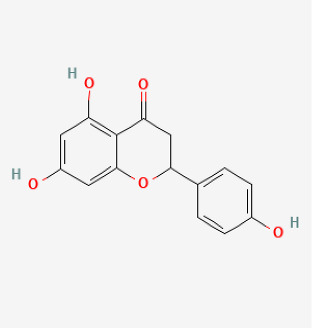	MCT rat	naringenin (50 mg/kg) were orally administered daily for 21 days	Alleviated oxidative stress, inflammatory and apoptotic markers.	[180]
Berberine	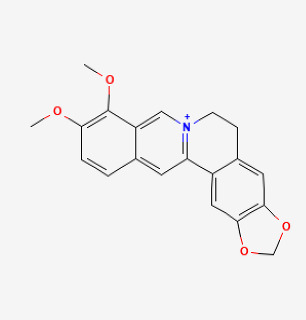	SuHx in rats	berberine (100 mg per kg day) for 28 days	Reversed right ventricular systolic pressure and right ventricular hypertrophy and decrease pulmonary vascular remodeling.	[181]
		MCT rat	berberine (50 mg/kg/d) for 28 days	Inhibited pulmonary artery smooth muscle cells’ proliferation and migration.	[182]
Tanshinone IIA	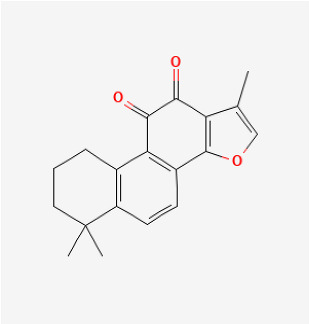	MCT rat	tanshinone IIA (10 mg/kg) for 14 days	Inhibited pulmonary artery intimamedia thickening and muscularization of the pulmonary arterioles.	[183]
		MCT rat	tanshinone IIA (30 mg/kg/d) for 21 days	Decreased RVSP, MRVP, RV/(LV+S) and pulmonary vascular remodeling.	[184]
		Hypoxia rat	tanshinone IIA (160 mg/kg/d) for 28 days	Inhibited proliferation of pulmonary artery smooth muscle cells.	[185]
		Hypoxia rat	tanshinone IIA (30 mg/kg/d) for 21 days	Normalized the RVSP and RVH, improved the cardiac output.	[186]
		Hypoxia rat	tanshinone IIA (30 mg/kg/d) for 21 days	Prevented hypoxia-mediated increases in intracellular calcium homeostasis and cell proliferation.	[187]
		MCT rat or Hypoxia mice	tanshinone IIA (10 mg/kg/d) for 21 days	Relieved RVSP and RVH.	[188]
		Hypoxia rat	tanshinone IIA (10 mg/kg/d) for 28 days	Restrained pulmonary artery wall remodeling.	[189]

## Data Availability

Not applicable.

## Abbreviations

PH	Pulmonary hypertension
Nrf2	Nuclear factor erythroid 2-related factor 2
VSMC	vascular smooth muscle cell
PAEC	pulmonary arterial endothelial cell
PASMC	pulmonary arterial smooth muscle cell
PAH	Pulmonary arterial hypertension
ROS	reactive oxygen species
NOS	nitric oxide synthase
NADPH	nicotinamide adenine dinucleotide phosphate
SOD	superoxide dismutase
ET-1	endothelin-1
PGI2	prostacyclin
EC	endothelial cell
SMC	smooth muscle cell
EMT	epithelial-mesenchymal transition
NO	nitric oxide
EDHF	endothelium-derived hyperpolarizing factor
VGCC	Voltagegated calcium channels
MCT	monocrotaline
NHE	Na^+^/H^+^ exchanger
ARE	antioxidant response element
EpRE	electrophile response element
GST	glutathione S-transferase
GCL	glutamate-cysteine ligase
GPx	glutathione peroxidase
TXNRD	thioredoxin reductase
TXN	thioredoxin
GSH	glutathione
sMAF	small musculoaponeurotic fibrosarcoma
cAMP	cyclic adenosine monophosphate
β-TrCP	β-transducin repeat-containing protein
RXR-α	retinoic X receptor alpha
Keap1	Kelch-like ECH-associated protein 1
BTB	Bric-a-Brac domain
IVR	intervening region
LncRNA	long non-coding RNA
RVH	right ventricular hypertrophy
SFN	sulforaphane
KCNK3	potassium channel subfamily K member 3
Ipt	Iptakalim
PPARγ	peroxisome proliferator gamma
DMF	Dimethyl Fumarate
RVSP	right ventricular systolic pressure
SuHx	SU5416 and 10% hypoxia
GCL	glutamate-cysteine ligase
RV/(LV+S)	right ventricular hypertrophy index
